# Oxidative Stress and Neurodegenerative Diseases: A Review of Upstream and Downstream Antioxidant Therapeutic Options

**DOI:** 10.2174/157015909787602823

**Published:** 2009-03

**Authors:** Bayani Uttara, Ajay V. Singh, Paolo Zamboni, R.T Mahajan

**Affiliations:** 1 Department of Biotechnology, M. J. College, M. J. Road, Jalgaon- 425 001, India;; 2 Centro Interdisciplinare Materiali e Interfacce Nanostrutturati (CIMAINA), Dipartimento di Fisica, Universita di Milano, Via Celoria 16, 20133 Milan, Italy;; 3 Centre for Vascular Disease, University of Ferrara, 41100 Ferrara, Italy

**Keywords:** ROS, oxidative stress, antioxidants, neurodegenerative diseases, rns, amyloid, catalase, phagocytes.

## Abstract

Free radicals are common outcome of normal aerobic cellular metabolism. In-built antioxidant system of body plays its decisive role in prevention of any loss due to free radicals. However, imbalanced defense mechanism of antioxidants, overproduction or incorporation of free radicals from environment to living system leads to serious penalty leading to neuro-degeneration. Neural cells suffer functional or sensory loss in neurodegenerative diseases. Apart from several other environmental or genetic factors, oxidative stress (OS) leading to free radical attack on neural cells contributes calamitous role to neuro-degeneration. Though, oxygen is imperative for life, imbalanced metabolism and excess reactive oxygen species (ROS) generation end into a range of disorders such as Alzheimer’s disease, Parkinson’s disease, aging and many other neural disorders. Toxicity of free radicals contributes to proteins and DNA injury, inflammation, tissue damage and subsequent cellular apoptosis. Antioxidants are now being looked upon as persuasive therapeutic against solemn neuronal loss, as they have capability to combat by neutralizing free radicals. Diet is major source of antioxidants, as well as medicinal herbs are catching attention to be commercial source of antioxidants at present. Recognition of upstream and downstream antioxidant therapy to oxidative stress has been proved an effective tool in alteration of any neuronal damage as well as free radical scavenging. Antioxidants have a wide scope to sequester metal ions involved in neuronal plaque formation to prevent oxidative stress. In addition, antioxidant therapy is vital in scavenging free radicals and ROS preventing neuronal degeneration in post-oxidative stress scenario.

## INTRODUCTION

Free radicals are molecules with unpaired electron in their outer orbit. Free radicals have very important role in origin of life and biological evolution, leaving beneficial effects on the organisms [57]. Oxygen radicals are involved in many biochemical activities of cells such as signal transduction, gene transcription and regulation of soluble guanylate cyclase activity. Nitric oxide (NO) is an important signaling molecule that essentially regulates the relaxation and proliferation of vascular smooth muscle cells, leukocytes adhesion, platelets aggregation, angiogenesis, thrombosis, vascular tone and hemodynamics [95]. Humans are constantly exposed to free radicals created by electromagnetic radiation from the manmade environment such as pollutants and cigarette smoke. Natural resources such as radon, cosmic radiation, as well as cellular metabolisms (respiratory burst, enzyme reactions) also add free radicals to the environment. The most common reported cellular free radicals are hydroxyl (OH·), superoxide (O_2_^–^·) and nitric monoxide (NO·). Even some other molecules like hydrogen peroxide (H_2_O_2_) and peroxynitrite (ONOO–) are not free radicals; they are reported to generate free radicals through various chemical reactions in many cases [30]. Cells exposed to environment fortified with oxygen continuously generate oxygen free radicals (OFR). Antioxidants defense systems co-evolved along with aerobic metabolism to counteract oxidative damage from OFR [92]. Human body produce oxygen free radicals and other reactive oxygen species as by products through numerous physiological and biochemical processes. Oxygen related free radicals (superoxide and hydroxyl radicals) and reactive species (hydrogen peroxide, nitric oxide, peroxynitrile and hypochlorous acid), are produced in the body, primarily as a result of aerobic metabolism [34, 66]. At the same time, antioxidants, such as glutathione, arginine, citrulline, taurine, creatine, selenium, zinc, vitamin E, vitamin C, vitamin A and tea polyphenols help to regulate the ROS thus generated. Antioxidant is further supported with antioxidant enzymes, e.g. superoxide dismutase, catalase, glutathione reductase and glutathione peroxidase those exert synergistic actions in removing free radicals [66, 81, 93].

Overproduction of free radicals can cause oxidative damage to biomolecules, (lipids, proteins, DNA), eventually leading to many chronic diseases such as atherosclerosis, cancer, diabetics, rheumatoid arthritis, post-ischemic perfusion injury, myocardial infarction, cardiovascular diseases, chronic inflammation, stroke and septic shock, aging and other degenerative diseases in humans [27, 93]. Excess NO is cytotoxic either by combining with tyrosine that is essential for catalytic function of enzyme ribonucleoside diphosphate reductase or by forming ONOOֿ. Excess vascular O_2_ֿ production could contribute to hypertension and vasospasm [39, 49, 60].

ROS are particularly active in the brain and neuronal tissue as the excitatory amino acids and neurotransmitters, whose metabolism is factory of ROS, which are unique to the brain and serve as sources of oxidative stress. ROS attack glial cells and neurons, which are post-mitotic cells and therefore, they are particularly sensitive to free radicals, leading to neuronal damage [30]. It has been reported that deleterious effects of ROS on human cells may end in oxidative injury leading to programmed cell death i.e. apoptosis [71].

Antioxidants are classified as exogenous (natural or synthetic) or endogenous compounds, both responsible for removal of free radicals, scavenging ROS or their precursors, inhibiting formation of ROS and binding metal ions needed for catalysis of ROS generation. [30].

Natural antioxidant system is sorted in two major groups, enzymatic and non- enzymatic. Enzymatic antioxidants are comprised of limited number of proteins such as catalase, glutathione peroxidase as well as superoxide dismutase (SOD) along with some supporting enzymes. Non-enzymatic antioxidants include direct acting antioxidants, which are extremely important in defense against OS. Most of them include ascorbic and lipoic acid, polyphenols and carotenoids, derived from dietary sources. The cell itself synthesizes a minority of these molecules. Indirectly acting antioxidants mostly include chelating agents and bind to redox metals to prevent free radical generation [30].

### Need of Antioxidants

It has been reported in epidemiological studies that many of antioxidant compounds posses anti inflammatory, antiatherosclerotic, antitumor, antimutagenic, anticarcinogenic, antibacterial and antiviral activities to greater or lesser extent [59, 64, 70]. In many cases, increased oxidative stress is a widely associated in the development and progression of diabetes and its complications which are usually accompanied by increased production of free radicals or failure of antioxidant defense [4, 5, 16, 18, 35, 58, 72, 91]. Though the intake of natural antioxidants has been reported to reduce risk of cancer, cardiovascular diseases, diabetes and other diseases associated with aging, there is considerable controversy in this area [38, 47, 77, 87]. Leukocytes and other phagocyte destroy bacteria, parasites and virus-infected cells with NO, O_2_, H_2_O_2_, and OCl, those are powerful oxidants and protect humans from infection. However, they cause oxidative damage and mutation to DNA and participate in the carcinogenic process if unchecked. In many cases, it is concluded that antioxidants modulate the pathophysiology of chronic inflammation up to some extent [54, 62, 73, 75, 76, 86]. Moreover, experiments and studies infer that antioxidants are needed to scavenge and prevent the formation of ROS and reactive nitrogen species (RNS); out of them, some are free radicals while some are not [3]. There is growing evidence that oxidative damage to sperm DNA is increased when there is ascorbate insufficiency in diet [8]. This strongly suggests the protective role of antioxidant in our daily diet.

### Sources of Antioxidants

Four endogenous sources appear to account for most of the oxidants produced by cells. (1) Normal aerobic respiration in which mitochondria consume O_2_, reduces it by sequential steps to produce O_2_, H_2_O_2_, and -OH as byproduct. (2) Bacteria or virus infected cells get destroyed by phagocytosis with an oxidative burst of nitric oxide (NO), O_2_^-^, H_2_O_2 _and OCl. (3) Peroxisomes produce H_2_O_2_ as a by-product of fatty acid and other lipid molecular degradation, which is further degraded by catalase. Evidence suggests that, certain conditions favor escape of some of the peroxide from degradation, consequently releasing it into other compartments of the cell and increasing oxidative stress leading to DNA damage. (4) Animal Cytochrome P_450_ enzymes are one of the primary defense systems that provides protection against natural toxic chemicals from plants, the major source of dietary toxins. Even these enzymes are protective against acute toxic effects from foreign chemicals, yet they may generate some oxidative byproducts that damages DNA [8].

Various antioxidants are supplied to human body through diet, both vegetarian as well as non vegetarian. Vitamins C and E, β-carotene and coenzyme Q are the most famous antioxidants of diet, out of which, Vitamin E is present in vegetable oils and found abundantly in wheat germ. It is fat soluble vitamin, absorbed in the gut and carried in the plasma by lipoproteins. Out if 8 natural state isomeric forms of vitamin E, α-tocopherol is the most common and potent isomeric form. Being lipid soluble, vitamin E can effectively prevent lipid peroxidation of plasma membrane [10, 11].

Plants (fruits, vegetables, medicinal herbs) may contain a wide variety of free radical scavenging molecules such as phenolic compounds (Phenolic acids, flavonoids, quinons, coumarins, lignans, stilbenes, tannins etc.), nitrogen compounds (alkaloids, amines, betalains etc.), vitamins, terpenoids (including carotenoids) and some other endogenous metabolites which are rich in antioxidant activity [15, 19, 83, 96].

## NEURODEGENERATIVE DISEASES AND OXIDATIVE STRESS

Neurodegenerative diseases comprise a condition in which nerve cells from brain and spinal cord are lost leading to either functional loss (ataxia) or sensory dysfunction (dementia). Mitochondrial (Mt) dysfunctions and excitotoxicity and finally apoptosis have been reported as pathological cause for aging and neurodegenerative diseases such as Parkinson’s disease (PD), Alzheimer’s disease (AD), Multiple Sclerosis (MS) and amyolotrophic lateral sclerosis (ALS). Neurodegeneration have been speculated to be interplay of a number of factors including environmental and genetic predisposition but redox metal abuse occupies central role as most of symptoms stems out from abnormal metal metabolism [53]. Oxidative stress and free radical generation catalyzed by redox metals have been shown to play pivotal role in regulating redox reactions *in vivo* contributing RNS and ROS, main culprits in neurodegeneration [42]. While considering role of oxidative stress in neurodegeneration, few important aspects need to be mentioned in brief are-

1. Oxygen is important for cellular function and oxygen exchange is normal phenomena for oxidative phosphorylation in Mt, then how it becomes malicious for neuronal cells?
2. Why neuronal cells particularly are most sensitive to oxidative stress?
3. What is the role of environmental and genetic factor in stimulating oxidative stress and subsequent neuronal cell death?
4. How does unregulated metabolism of redox metal comes in picture in generating oxidative stress?

### Oxygen and Oxidative Stress

Oxygen is vital for all living cells whether neuronal or other kinds of cells taking part in tissue formation but on the other hand it is potentially dangerous in excess. Thus, it is kept under tight check of complex system that regulates and monitors the usage and uptake of this essential element. Oxygen takes part in glucose break down in Mt through oxidative phosphorylation and generates energy currency of cell i.e. ATP [36]. Mt has its own molecular machinery (Mt DNA) for synthesis of enzyme and proteins required for oxidative phosphorylation. Any mutation in Mt DNA leads to impaired ATP generation and perturbed oxidative phosphorylation cascade that may further lock the neuronal function [33]. Oxidative stress arises due to disturbed equilibrium between pro-oxidant/antioxidant homeostasis that further takes part in generation of ROS and free radicals those are potentially toxic for neuronal cells. The reason for neuronal cell hypersensitivity towards oxidative stress arises due to anatomic and metabolic factors. In the brain, various types of glial cells are present and these are involved in anatomic support and metabolic requirement. The endothelial cells surrounding these glial cells are less permeable for uptake of various molecules and protective cells viz. macrophages compared to other endothelial cells in the body. In addition, glial cells in brain require more oxygen and glucose consumption to generate continuous ATP pool *in vivo* for normal functioning of brain as it is one of busiest organ to keep all other organs active and under control. That makes them more susceptible towards oxygen over load, thus free radical generation [49]. Under physiological condition, 1-2% of O_2_ consumed is converted to ROS but in aged brain this percentage goes up due to reduced surveillance of antioxidants and low regenerative capacity of aged brain [49].

### ROS: Real Culprits for Neuronal Degeneration

ROS comprises hydrogen peroxide (H_2_O_2_), nitric oxide (NO), superoxide anions and the highly reactive hydroxyl and monoxide radicals (OH·, NO·). Damaged Mt and activated microglia acts as reservoir of ROS. Initially ROS generation was believed to be an outcome of imbalance between generation and elimination of ROS and RNS but recently many chemistries and molecular biology have been discovered regulating ROS those play fundamental role in modulating key cellular functions [46]. For example, Haber Weiss and Fenton reaction initiate the free radical and ROS generation that activates mitogen activated protein (MAP) kinase cascade, excitotoxic calcium mobilization and finally apoptotic cell death [40]. Free radicals have been reported for their great contribution to neuronal loss in cerebral ischemia, seizure disorders, schizophrenia, Parkinson's disease and Alzheimer's disease [14, 21, 67, 68, 79, 89, 90].

### Pathological Evidences of ROS Mediated Neuronal Damage

Neuronal biochemical composition is mainly susceptible to ROS since it involves pool of unsaturated lipids those are labile to peroxidation and oxidative modification. Double bonds of unsaturated fatty acids are hot spots for attack by free radicals those initiate cascade or chain reaction to damage neighboring unsaturated fatty acids [13]. Several researchers considered brain to be abnormally sensitive to oxidative damage and many studies demonstrative of the ease of peroxidation of brain membranes supported this notion [17, 24, 94]. Brain contains high level of fatty acids which are more susceptible to peroxidation, that consumes an inordinate fraction (20%) of total oxygen consumption for its relatively small weight (2%). In addition, it is not particularly enriched in antioxidant defenses. Brain is lower in antioxidant activity in comparison with other tissues, for example, about 10% of liver. Moreover, human brain has higher level of iron in certain regions and in general has high levels of ascorbate. As evident from above data, neural cells are considered to be more susceptible to oxidative damage as compared to other body tissues [24].

### Mechanism of ROS Generation

ROS generation is prerequisite of metabolic system in order to interact with organic molecules *in vivo* as interaction of organic molecules with oxygen is energetically unfavorable. In all forms of ROS generation, molecular oxygen needs to be activated and cellular system have evolved range of metallo-enzymes those facilitates ROS generation upon interaction of redox metals with O_2 _using various catalytic pathways. Since free radicals are toxic to cells, under normal circumstances, cells have efficient regulating system for O_2 _and metal ion interaction leading to free radicals and ROS generation [12].

Fe^3+^ + •O_2_^−^ → Fe^2+^ + O_2 _(Step I)

Fe^2+^ + H_2_O_2_ → Fe^3+^ + OH^−^ + •OH (Step II) *

Combining step I&II

•O_2_^-^ + H_2_O_2_ → •OH + HO^-^ + O_2 _* Known as Fenton reaction [69]

A part from direct ROS generation, there are different *in vivo* pathways those contribute substantively ROS generation by calcium activation with metallo-enzymes. Calcium is an important signaling molecule and it is required for many cellular responses and cell-cell communication. Thus, any disturbance in stimulus and regulation of calcium pathway may disrupt the cellular physiology [1].

### Mechanism of ROS Mediated Cellular Apoptosis

As evident from terminology, ROS are extremely reactive to different fundamental molecules in cellular pool and initiate cascade of reactions at same time that leads to neuronal cell death. Oxidative over load in neuronal microenvironment causes oxidation of lipids, proteins and DNA and generates many byproducts such as peroxides, alcohols, aldehydes, ketones and cholesterol oxide. Most of them are toxic to blood lymphocyte and macrophages, paralyzing the *in vivo* defense system [23]. Cystine, lysine and Histidine residues in protein are hot spot for acrolein (oxidatively modified lipid) and NHE (Sodium Hydrogen Exchanger) for modification and they cross link these amino acid residues *via* Michael addition as given below [41]. 


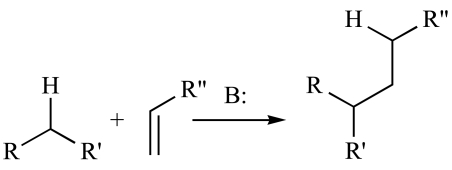


Where B is the Base e.g. NaOH, KOH etc.

Acrolein hampers glutamate and sugar uptake where as NHE block neuronal ion transporters and activates c-Jun and MAP kinase pathways to invoke cellular apoptosis [45]. Protein modification leads to loss of function of enzymes regulating oxidative balance in cellular system viz. glutamine synthase, superoxide dismutase. Most significant ill effect on neuronal health takes place by dysregulation of intracellular calcium signaling pathways initiated by ROS and in recent years heavy evidences suggest role of ROS in neuronal cell death [22]. Excitotoxic effects initiated by ROS induced intracellular calcium influx leads to activation of glutamate receptors and apoptosis in Huntington’s, AD, PD and ALS. In addition, DNA mutations add further insult after ROS mediated modifications [55].

### Metallobiology and Neurodegenerative Diseases (AD, PD, MS and ALS)

Most astounding effect of aging can be described as neurodegeneration associated with disturbed metal metabolism. In aged brain, accumulation of redox metals (Copper, Iron and Zinc) have been found to substantively increased due to concentration of metals by blood brain barrier (BBB) at junction of neuronal environment and blood vessels. This makes aged brain more prone to initiate neurodegeneration in vicinity of neuronal cells in brain [78].

AD is characterized by Amyloid plaques deposition by chelating Amyloid-β peptide (Aβ) with transition metal ions (Cu^2+^, Zn^2+^and Fe^3+^). Toxicity of Aβ is attributed due to histidine residues at position 6, 13 and 14 those are structural site for transition metal coordination. Binding of Cu^2+^and Fe^3+^produces toxic chemical reaction, altering oxidation state both the metals, producing H_2_O_2 _catalytically in presence of transition metals and finally gives toxic OH^**˙**^free radicals [63]. One interesting aspect about Aβ plaques is that researchers consider Aβ plaques as toxic species responsible for AD but latest reports suggest Aβ as a physiological antioxidant and this property is modified due to aging. In future this property may be used as potent therapy for AD [20].

PD is characterized by deposition of inclusion bodies (Lewy bodies) of α-synuclein in substantia nigra that is ubiquitously expressed in brain and mutation in this principle protein is reported in familial forms of AD [28]. Dopamine has a good reputation as neurotransmitter but at the same time it is very good metal chelator and electron donor that set *in vivo* conditions for redox metal chemistry to generate toxic free radicals. It has high tendency to coordinate with Cu^2+ ^and Fe^3+ ^and reducing metals to initiate Fenton’s chemistry to generate H_2_O_2_ [29]. Evidences indicate that mutations in α-synuclein protein has a role in modulating the dopamine activity but in negative way that initiates neuronal cytoplasmic accumulation and interaction of dopamine with iron, engendering ROS production [51]. In addition, mutations in α-synuclein support various intracellular pathways those dysregulate dopamine-metal interactions and ROS generations. An example is loss of neuromelanin cell from substantia nigra in PD patient. Neuromelanin is dark brown pigment with unknown function but strong evidence suggest that it accumulates redox metals in aged brain and supposed to be product of dopamine redox chemistry [84].

In MS and experimental allergic encephalomyelitis (EAE), an animal models of MS, have been characterized as autoimmune neuronal disorder that causes demylination of central nervous system (CNS). Unregulated iron metabolism and ROS generation have been named as major player in pathogenesis of disease. High lipid content generated by myelin and oligodendrocytes invite massive accumulation of iron and other metals since redox metals acts as catalytic center for this lipid factory. Iron plaque deposited over myelin sheath invokes an inflammatory response that triggers recruitment of inflammatory cell such as tissue macrophage and T cells entering into CNS to cause substantive damage and demyelination to CNS [80].

In ALS, like MS, lower motor neurons from spinal cord and cerebral cortex are lost due to deposition of a misfolded protein in neuronal tissue in relation with toxic gain of function by mutated sulfur oxide dismutase (SOD) enzyme associated with Cu/Zn redox metallobiology [52]. Gain in toxic function in mutated SOD is due to loss of active sites for Cu binding that leads to conversion of SOD itself in pro-oxidant protein that participates in ROS generation [82].

Apart from aforementioned neuronal disorders, In Friedreich’s ataxia, loss of mitochondrial protein frataxin is caused by abnormal iron accumulation and ROS generation. In addition, iron overload causes mitochondrial respiratory chain breakdown and oxidative stress leading to cardiomyopathy and neurodenegeration [7].

### Genetic Evidences in Neurodegenerations and Oxidative Stress

Oxidative stress related neurodegeneration is not only caused by disturbed metal metabolism but genetic evidences suggests that persons associated with certain types of genetic mutations are more susceptible to gain neurological pathological compare to normal to those with normal genetic profile. Person with hemochromatosis (HFE) associated mutations may be on higher side of developing iron over load related oxidative stress and neuropathology with ingestion of daily iron supplement [31,37]. Metal metabolism is combined interplay between genes related with synthesis of metalloenzymes and dietary metal supplement. Any imbalance in this interaction favors dysregulated cellular metallobiology that subsequently leads to neurodegenerations. Clinicians suggests it is made to be mandatory to counsel the patients with associated mutations and increased risks of neurodegeneration.

### Antioxidant Therapeutic Option to Upstream of OS: Enzymes and Antioxidants Dedicated to Regulate Protein-Transition Metal Interaction and Reduce Free Radical Generation

Antioxidants are exogenous or endogenous molecules those act against any form of oxidative stress and its associated ill effects on cellular system. They neutralize ROS and other kinds of free radicals produced as consequence of OS and have attracted the attention of clinicians due to therapeutic potential. Thanks to our daily diet that contains millions of natural antioxidants in form of flavonoids and phenolic compounds, lipoic acid (thioctic acid), ubiquinone and idebenone, β-carotene and vitamin C as metabolic supplements and keeps all our vital organs free from OS [49].

As stated above the prime focus of antioxidant therapy should be to interrupt and modulate the neuronal protein interaction with culprit redox metals instead of therapeutic approaches surrounding downstream effect of ROS. Thus, antioxidant therapy preventing corruption and breakdown of metalloenzyme and innate antioxidant defense system supporting proportional metal homeostasis will gain clinical appraisal. So for, hallmark therapeutic strategies clinically target the relevant transition metals, those take part in neurodegeneration. In certain cases, anti-inflammatory drugs are given as supplement at onset of neurological disorder to pacify immune system as in certain circumstances; immune system is significantly provoked due to oxidative stress. Antioxidant therapy involving enzymes and anti-inflammatory drugs constitute upstream therapy in ROS generation and prevent downstream pathologies in advance in certain neurodegeneration. Oxyradicals have a very short life (1 µs) and usually are inactivated or scavenged by antioxidants before they can inflict damage to lipids, proteins or nucleic acids. In this regard, pathology due to ROS can be checked by at least two mechanisms

Inactivation of oxyradicals by dietary antioxidants like vitamin C, vitamin E, β-carotene.

Replacement of esterified membrane phospholipids with polyunsaturated fatty acids (PUFAs) by dietary supplementation with essential fatty acids [10].

### Dietary Natural Antioxidants as Upstream Preventive Measure

There are clinical evidences that neurodegenerations can be ameliorated upon dietary intake or supplementary intake of natural antioxidants. Dietary intake contains variety of antioxidants vitamin supplements those play a vital role in neuroprotection in variety of neurological disorders [65].

These natural antioxidants prevent oxidation of proteins, lipid peroxidations and prevent generation of ROS, thus act as upstream therapeutic barrier to OS.

One of important futuristic upstream therapeutic aspect that can regulate oxidative stress to protect neuronal cells from death is vaccination against potential toxic protein formed in different types of neuronal disorders. A promising example is Amyloid-β vaccination in AD that prevents plaque formation and subsequent neuron inflammation [43]. This could be a therapeutic strategy for other neurological disorders lead by OS, such as in MS.

### Downstream Antioxidant Therapy in ROS Mediated Neuronal Disorder: Preventing Neuronal Inflammation and Free Radical Scavenging

Point of ROS generation and past events embark a number of side reaction those directly or indirectly initiate neuronal interaction. Thus, there must be therapeutic coverage for such post oxidative stress events. A number of natural and synthetic products have been advised to work in neuroprotection to combat the ill effects oxidative stress. Ginkgo biloba (EGb 761), famous Chinese herb has been known for its excellent antioxidant properties that restrict β-amyloid toxicity after plaque formation [88]. In mild AD patients, it is shown that EGb 761 improves cognitive activities and neuronal function though in severe AD, neuroprotective role of EGb 761 seems to be reduced. Inflammatory reactions are most common features of all forms of neuronal disorders. Non Steroidal Anti-inflammatory Drugs (NSAIDS) are most praised downstream therapeutics, ameliorating inflammatory infiltration of macrophages. They act as antioxidants to reduce inflammatory cascade induced by oxidative stress [25]. CPI-1189, a nitrone related compound is supposed to down-regulate the pro-inflammatory cytokine cascade of genes in primary glial cells. In this regard, nitron and related compounds are under phase III clinical trial for potential commercial applications [26]. In addition, *in vivo* proteins and growth factors such as brain derived neurotrophic factors, responsible for enhancing memory and cognitive function in OS, can be induced in response to promote growth and survival of deteriorating neurons [56].

Another chemical moiety that resembles vitamin E in its chemical structure is female sex hormone estrogen (estradiol) that contains a phenolic free radical scavenging site and acts as anti oxidant. Interestingly, it posses capability to prevent an upstream neurodegeneration and downstream the oxidative overload. For example, 17 -Estradiol prevents neuronal cell death either inhibiting H_2_O_2 _formation or preventing Aβ toxicity, the 2 events taking place before and after of oxidative stress respectively. Antioxidant properties of these phenolic compounds are due to interaction of their functional group with the redox metals, not on account of their cellular oestrogen receptors [32]. Table**1** gives details of prominent antioxidant with their class, mechanism of action and referral of neurodegenerative disorder.

### Modulation of Calcium Mediated Excitotoxic Effects as Therapeutic Options

Disruption of homeostatic metal metabolic pathways by ROS leads to increased intracellular calcium levels that cause neuronal cell death due to dysregulated microtubules assembly and axonal transport. *In vitro* studies had shown the capabilities of natural oxidants such as Taxol in preventing neuronal cell death by preventing microtubule disaggregation [9]. This gives vital downstream therapeutic target to prevent calcium mediated neurotoxicity in clinical AD, PD and ALS.

### Challenges in Designing Pre and Post Oxidative Stress Antioxidants Therapeutics

The major challenge in designing synthetic antioxidant to protect brain cognitive function due to neuronal death is movement across blood brain barrier (BBB). Chemical opening due to osmotic differential pressure at BBB tight junction or pathological openings due to trauma or pathogenic causes are the means to molecular motion across BBB. Coenzyme Q10 (ubiquinone), GSH and oxidized form of vitamin C have shown substantive ability to cross BBB in human and rodent models [30]. Thus, designing artificial antioxidant drugs for neuroprotection must accompany aforementioned structure analogue in order to cross BBB and circulate in brain neuronal circuitry.

### Concluding Remarks and Future Perspective

It is clear from current neurobiology research that unregulated metal metabolism plays catastrophic role in catalysing *in vivo* chemical reactions those lead to oxidative stress and neuronal cell death as final cause. Though metals are crucial as cofactors to carry out numerous *in vivo *catalytic enzymatic reactions in cellular metabolism and cell signalling. Any mutations in Mt DNA or metal overload in aged brain leads to oxidative stress and free radical mediated pathological changes in neurons. Neuronal proteins and structural components get modified due to OS in different neurological disorders leading to neuro-inflammation and loss of cognitive function in AD, PD, MS and ALS. Since in this review, OS have been defined as principle pathological cause of neurodegeneration, antioxidants are proposed as therapeutic options to combat the free radical generation and maintenance. This review covers the sources of antioxidants and free radicals and general mechanism involve in antioxidant mediated free radical scavenging. Major emphasis have been given on the role of oxidative stress and free radical chemistry with respect to major neurodegenerative disorders viz. AD, PD, MS and ALS. We report major antioxidant therapeutic target those are capable in neuroprotection before OS (upstream), majority preventing free radical generation, modulating metal-neuronal protein interaction and promoting normal metal homoeostasis. In additions, we have given an account of antioxidant in post OS (downstream) taking care of neuronal inflammation and free radical scavenging with challenges in designing therapeutic antioxidants.

## Figures and Tables

**Fig. (1):**
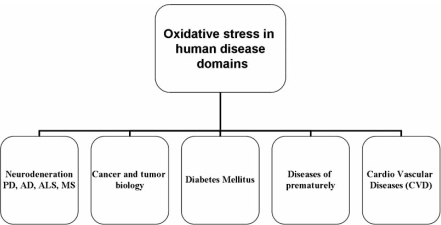
show human diseases where oxidative stress plays direct or indirect role in pathophysiology of disease.

**Fig. (2):**
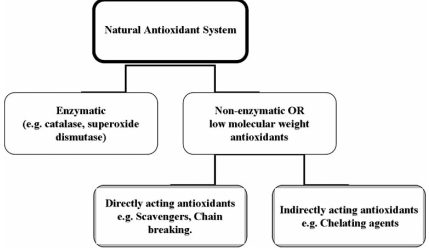
shows a comprehensive categorization of natural antioxidants.

**Fig. (3):**
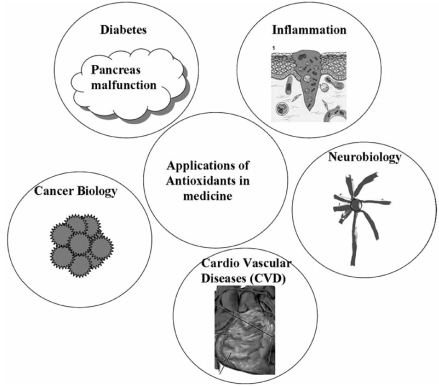
depicts vital applications of antioxidants in medicine and biology.

**Fig. (4):**
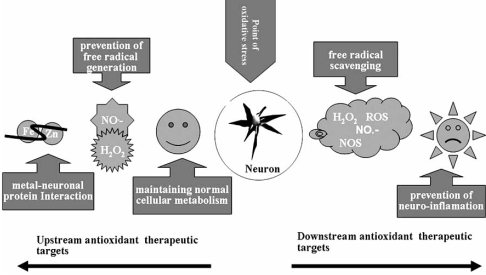
defines strategic antioxidant therapeutic targets before (up) and after (down) oxidative stress leading to neuronal degeneration.

**Table 1:** Details of Prominent Antioxidant with their Class, Mechanism of Action and Referral of Neurodegenerative Disorder

Class	Chemical Composition	Mechanism of Action	Neuronal Disorder Studied	Reference
Direct Antioxidant	Aryl amines and indoles-carotene, lycopene Polyenes- carotene, lycopene, retinol Selenium containing compounds ebselen. Polyphenols- Flavonoids, stilbenes, and hydroquinone Monophenols: tocopherols(vitamin E), 17-estradiol (estrogen), 5-hydroxytryptamine (serotonin)	direct chemical (nonenzymatic)scavenging of ROS generated free radicals and get some of them are recycled with endogenous oxidoreductases or via intracellular reducing shuttles	neuroprotection in Alzheimer’s disease	6, 37
Indirect antioxidant	Amino oxidase inhibitors, calcium antagonists, dopamine receptor agonists, glutamate receptor antagonists, ion chelators, nitric oxide synthase inhibitors	Prevent excitotoxicity, ROS and free radical generation targeting inhibitors and receptors dysregulating metal homeostasis	Parkinson’s disease and dementia	37, 84
Metabolic antioxidant	N-acetyl-cysteine, glutathione, 2-oxo-thiazolidine-4-carboxylate, and other thiol-delivering compounds N-butyl- -phenylnitrone. Carnitine, creatine, lipoic acid (thioctic acid), ubiquinone and idebenone	nullify cellular damage caused by free radicals by reducing secondary metabolic burden over cellular organelle(Mt, vesicles and cell membrane	Neuroprotection in general	44, 2
Metal containing antioxidant	Manganese-containing mimetics of catalase/superoxide dismutase	Enzymatic mimicking prevents protein-metal interaction	Mt protection in AD and PD	73
